# Effects of physical exercise on working memory in older adults: a systematic and meta-analytic review

**DOI:** 10.1186/s11556-021-00272-y

**Published:** 2021-09-17

**Authors:** Cai Zhidong, Xing Wang, Jilin Yin, Dehai Song, Zhitong Chen

**Affiliations:** 1 Shanghai University of Sport, School of Physical Education and Training, Shanghai, 200438 China; 2 Physical Education Department, Beibu Gulf University, Qinzhou, 535011 China; 3 Institute of P. E Yancheng Teachers University, Yancheng, 224002 Jiangsu Province China; 4 School of Physical Education and Health, Shanghai Lixin University of Accounting and Finance, 2800 Wenxiang Road, Shanghai, 201620 China

**Keywords:** Physical exercise, Working memory, Older adults, Randomized controlled trial, meta-analysis

## Abstract

**Background:**

This systematic and meta-analytic review aimed to investigate the effects of physical exercise on the working memory of older adults, and to identify the moderators of these effects.

**Methods:**

We searched six electronic databases for randomized controlled trials on the effects of physical exercise on working memory that were published before or on May 15, 2020. The PEDro scale was used to evaluate the methodological quality of the included studies. Stata 14.0 software was used to perform the meta-analysis, subgroup analysis, and publication bias testing.

**Results:**

A total of 28 studies and 2156 participants were included. The methodological quality of the included studies was fair to excellent, and there was no publication bias. Overall, we found that physical exercise had a significant effect on working memory in older adults (standardized mean difference = 0.30, *p* < 0.0001). The effects of physical exercise on working memory were moderated by exercise frequency, intensity, type, duration, cognitive status, and control subgroup (active/passive), but not by intervention period or age of participant.

**Conclusion:**

Physical exercise can effectively improve the working memory of older adults. The recommended physical exercise is multi-component exercise or mind–body exercise of moderate intensity for 45–60 min 3 times a week, for more than 6 months.

**Supplementary Information:**

The online version contains supplementary material available at 10.1186/s11556-021-00272-y.

## Background

Working memory (WM) refers to a system in which individuals temporarily store and manipulate information during complex cognitive tasks [1]. WM is considered to be a core cognitive function, because it underlies the brain’s ability to simultaneously store and manipulate information. WM is closely related to activity of the frontal and parietal networks, and the prefrontal cortex (PFC) in particular is considered to be an important brain area involved in WM [2]. Within the brain network, PFC is associated with the executive processing components, while the medial temporal cortex and hippocampus are associated to encoding and retrieval [3]. Parietal brain regions are associated with the temporary storage components [4], where the integration of visuospatial and associative information takes place [5].

WM is a core cognitive function. Age-related neural changes in brain networks results in a WM performance decline with increasing age. The best WM performance has been reported to be at about the age of 30 years, and to decrease significantly after the age of 60 years [6]. Both human and animal studies have found that PFC activity decreases with age [7, 8]. Mattay et al. found that while there was no difference in the performance of the 1-back task between younger and older people, the older group exhibited more activation in the bilateral frontal cortex; that study also found that older people performed worse on the 2-back task than younger people, and this was accompanied by less PFC activation [9].

An increasing amount of research has shown that physical exercise can improve cognitive functioning. This is especially true for executive functioning, which is closely related to frontal lobe activity [10]. Physical exercise is considered to be a safe treatment option for WM decline [11]. In a randomized controlled trial (RCT) with 120 older adults, Erikson et al. found that aerobic exercise (AE) training increased the size of the anterior hippocampus, and that this was associated with improvements in spatial memory [12]. Ikudome et al. found that even simple resistance exercise (RE), which uses only body mass for resistance, may be an effective method for preventing the age-related cognitive decline of inhibitory control and WM in older people [13]. In another study, Yang et al. allocated 52 older women into a Tai Chi Chuan group, square dancing group, and control group. After the 6-month intervention period, the reaction time and accuracy rate of the n-back task in the Tai Chi Chuan and square dancing groups improved alongside a P3 amplitude increase and latency decrease, which indicated that the Tai Chi Chuan and square dancing interventions improved the WM of older women [14]. Weuve et al. found that higher levels of activity were associated with a better backward memory span, and also observed less cognitive decline among more active women [15]. Hatta et al. examined the effects of physical activity on WM (which was measured using the Sternberg task) in older adults, and found both behavioral and neurophysiological evidence for the positive role of exercise [16]. Namely, the high exercise group had significantly faster reaction times and a larger P3 amplitude than the low exercise group, but there was no significant between-group difference in latency. Chang et al. used the same research design and found that the high exercise group had a significantly larger N1 amplitude than the low physical activity group.

However, some studies have revealed different results. Kramer et al. found no improvements in the accuracy of n-back task or in digit span test (DST) performance after an AE intervention [17]. Gothe et al. found that the reaction time and accuracy of the n-back task after 20 min of yoga were better than those observed after moderate-intensity AE [18]. One reason for the inconsistency of these research results may be the variability in the exercise features (e.g., frequency, intensity, duration, type, and intervention period), which could engage mechanisms underlying WM improvements in different ways. Another potential reason for these different research results is the use of different WM measurement tools, such as the DST, n-back task, and Sternberg task. WM is a complex advanced cognitive function. Baddeley has argued that WM consists of at least three parts – central execution, phonetic loop, and visual-spatial storage [19] – which involve multiple processes, such as encoding, maintenance, updating, attention, and inhibition. Each measurement tool assesses different sub-components of WM. Thus, it is difficult to comprehensively investigate the intervention effect of physical exercise on WM using one single paradigm. Finally, individual differences such as age, cognitive status, and education level will also affect the efficacy of an intervention.

Previous meta-analyses have paid little attention to the intervention effect of physical exercise on WM, especially in older adults. The populations included in these reviews were either patients with Parkinson’s disease and schizophrenia [20] or healthy older adults [21]. No meta-analysis has considered participants with normal cognition and patients with mild cognitive impairment (MCI) at the same time. Some reviews have indicated that age, the type of WM test, and exercise intensity moderate the relationship between physical exercise and WM. However, these prior reviews have offered relatively little information about the optimal prescription of physical exercise features for improving WM [22, 23]. To address these gaps in the literature and provide a theoretical basis for accurate exercise prescription, this study analyzed the effects of exercise interventions on WM and examined whether these effects are moderated by variations in the features of physical exercise.

## Methods

This study was performed and reported according to Preferred Reporting Items for Systematic Reviews and Meta-Analyses [24]. We pre-registered our meta-analytic review at PROSPERO (CRD42021230431).

We searched six electronic databases (PubMed, Embase, The Cochrane Library, Web of Science, PsycINFO, China National Knowledge Infrastructure) from inception to April 13, 2020. According to the reviewer’s suggestion, we conducted a new literature search on April 12, 2021. Two researchers (CZD and YJL) independently used the following search terms (among others) for retrieval: “exercise”, “physical activity”, “fitness”, “aerobic exercise”, “cardiovascular exercise”, “resistance training”, “stretching”, “mind–body exercise”, “flexibility exercise”, “cognitive function”, “executive function”, “working memory”, “old people”, “old adults”, “randomized controlled trial”. The retrieval strategy adopted the combination of subject words and free words, and was determined after repeated prechecking. Language and publication types were not limited in the literature retrieval step.

**Table:** 

Search strategy of Pubmed #1 physical exercise OR physical activity OR exercise OR fitness OR training OR aerobic exercise OR cardiovascular exercise OR resistance training OR stretching OR mind-body exercise OR flexibility exercise #2 working memory OR cognitive task OR executive function OR executive control OR updating OR short-term memory #3 old people OR elderly OR old age OR the aged OR senior citizen #4 randomized controlled trial OR controlled clinical trial OR RCT OR clinical intervention #5 #1 AND #2 AND #3 AND #4	

## Eligibility criteria

Two researchers (CZD and YJL) independently screened the literature according to the inclusion and exclusion criteria. After the screening, any discrepancy between the two researchers was resolved through discussions with the other two researchers (SDH and YJL) until consensus was reached.

The inclusion criteria were as follows: (1) the subjects were older adults; (2) the intervention was AE, RE, multi-component exercise (MCE), or mind–body exercise (MBE); (3) all or some of the outcome indicators were WM; (4) the study was an RCT.

We set the following exclusion criteria: (1) the subjects were older adults with dementia or mental disorders; (2) the intervention program contained confounding factors other than exercise, such as cognitive training, vitamin supplements, and drugs; (3) the study data could not be extracted, even after contacting the authors; (4) publications that were qualitative studies, case studies, reviews, non-intervention studies, or conference papers.

### Data extraction

Two researchers (CZD and YJL) independently extracted the relevant information using a standardized form. Where data were missing or could not be extracted due to insufficient statistical reporting, we contacted the author(s) to request the missing data.

Extraction contents and coding were as follows. First, we captured the basic details of each study, including the names and nationalities of authors and the year of publication. Second, we collated and processed the basic details of the subjects, including cognitive status, sample size, age, and education level. Third, we captured data on the five following exercise prescription variables: frequency, intensity, duration, type, and intervention period [23]. Exercise frequency was classified according to the number of exercise sessions per week, as follows: low frequency: ≤ 2 times; moderate frequency: 3–4 times; high frequency: ≥ 5 times. Exercise intensity was classified as low, moderate, vigorous. Exercise type was classified as AE, RE, MCE, or MBE. Exercise duration (the minutes each session lasted) was classified as follows: short: ≤ 45 min; moderate: > 45 min to ≤60 min; long: > 60 min. Intervention period was classified according to the length of the intervention period, as follows: short: 4–12 weeks; mid-length: 13–24 weeks; long: > 24 weeks. Fourth, the control group was classified as follows: active control subgroup (who participated in stretching, health education, and/or social assembly) and passive control subgroup (who received no intervention). Finally, the main outcome index was DST result, and the secondary outcome indexes were the n-back, verbal span, Corsi block-tapping, executive control (EC), spatial span (SS), and letter-number sequence tasks. All behavioral measures of WM were extracted in the form of the mean and standard deviation.

### Assessment of study quality

Methodological quality was independently evaluated by two researchers (CZD and YJL) using the Physiotherapy Evidence Database (PEDro) scale [25]. The PEDro scale comprises the 11 following items: eligibility criteria, randomization, concealed allocation, similar baseline, blinding of subjects, blinding of therapists, blinding of assessors, more than 85% retention, intent-to-treat analysis, between-group comparison, point measure, and measures of variability. The “eligibility criteria” item is not scored. One point is assigned to each item for which relevant information is explicitly presented, and the maximum score for any given study is 10 (9–10 = excellent quality, 6–8 = good quality, 4–5 = fair quality, < 4 = poor quality).

### Statistical analysis

Stata 14.0 software (Stata, Texas, USA) was utilized for data analysis. Extracted data included the mean (M) and standard deviation (SD) of each group at postintervention, and the sample size. The standardized mean difference was selected as the magnitude of effect sizes (ESs). ESs were calculated by Cohen’s *d*, taking 0.2, 0.5, and 0.8 as the respective thresholds for small, medium, and large effects [26]. Heterogeneity was calculated using Higgins’s *I*^*2*^ statistics, taking 75, 50, and 25% as the respective thresholds for high, medium, and low ratios of inter-study heterogeneity [27]. Publication bias was tested using the Egger test in Stata 14.0.

After calculating the overall ES for WM, subgroup analyses were conducted for the measures of WM (e.g., DST-Backward (DSB), DST-Forward (DSF), n-back, and spatial span tasks), exercise prescription features (frequency, intensity, type, duration, length), and participant characteristics (age, control group, and cognitive status). We provided Forest plots of subgroups. Funnel plots of the ES against the standard error of the ES were visually inspected for small-sample bias, and Egger’s test values with 95% confidence intervals for funnel plot asymmetry were calculated.

## Results

### Literature search

**Fig. 1:**
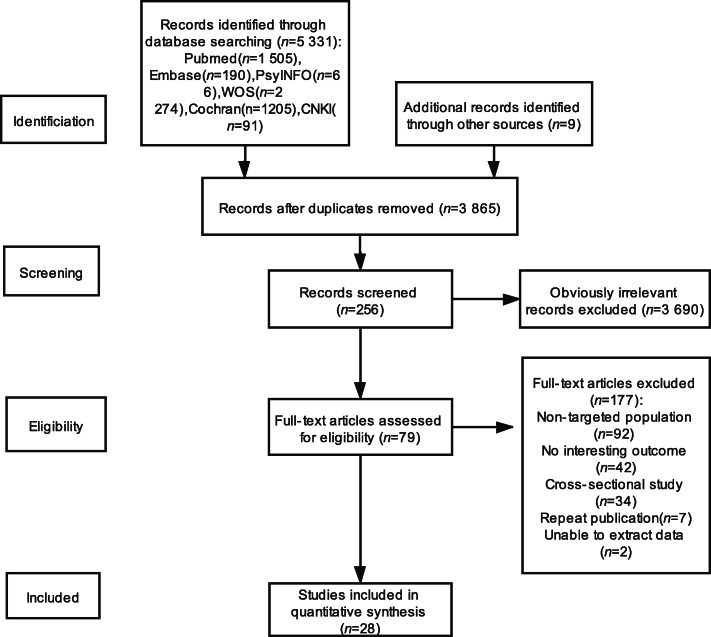
Literature Selection Flow Diagram

Figure 1 summarizes the flow of the literature search and study selection. The initial search returned 5340 articles. After removing 1475 duplicate articles and 3690 articles according to the inclusion/exclusion criteria and abstract screening, 28 articles were finally included in this review.

### Study characteristics

Table 1 presents the characteristics of all 28 studies included in this review. The sample size ranged from 19 to 210. The overall sample size was 2063, including 1016 participants in the experimental groups and 1047 in the control groups. Among the 28 studies included, participants of 11 articles were patients with MCI, and participants of 17 articles were normal older adults. Participants’ age ranged from 62 to 86 years. Participants were mainly female, except the Norouzi et al. study which only included men as the research subjects, Liu-ambrose and Damirchi only included women as the research subjects, and the remaining studies had no sex-based restrictions. The studies were performed in 16 countries, including Asian countries (16 papers, accounting for 57.1%), America (5 papers, accounting for 17.9%), European countries (5 papers, accounting for 17.9%), and Australia (2 papers, accounting for 7.1%).

**Table 1:** Basic characteristics of the literature included in the study(*M* ± *SD*)

Study	Country	Cognitive status	Sample(E/C)	Age(E/C)	Female proportion(E/C)	Education level(E/C)
Brown 2009a [49]	Astralia	N	66/34	79.5 ± 5.9/78.1 ± 6.4	86.6/86.8	10.6 ± 2.4/10.3 ± 2.3
Brown 2009b [49]	Astralia	N	26/34	81.5 ± 6.9/78.1 ± 6.4	91.6/86.8	10.2 ± 1.9/10.3 ± 2.3
Eggenberger 2016 [28]	Switzerland	N	19/14	72.8 ± 5.9/77.8 ± 7.4	63.2/64.3	13.4 ± 1.8/13.6 ± 2.1
Gothe 2016 [62]	America	N	61/57	C62.1 ± 5.8/62.0 ± 5.4	80.3/75.4	>college degree 77/56.2
Albinet 2016 [29]	America	N	19/17	67 ± 5/66 ± 5	68.4/76.5	11.9 ± 3.9/11.6 ± 2.1
Kalbe 2018 [50]	German	N	18/17	68.2 ± 8.0/67.5 ± 5.9	61.1/58.8	14.4 ± 3.5/14.5 ± 2.9
Liu-ambrose 2010a [37]	Canada	N	46/47	69.4 ± 3.0/70.0 ± 3.3	100/100	>high school 98.2/98
Liu-ambrose 2010b [37]	Canada	N	42/47	69.5 ± 2.7/70.0 ± 3.3	100/100	>high school 98.2/98
Hariprasad 2013 [43]	India	N	60/60	75.74/74.78	58.1/62.1	13.1 ± 4.1/11.4 ± 4.4
Norouzi 2019 [38]	Iran	N	20/20	68.3 ± 4.1/68.1 ± 3.7	0/0	NR
Nouchi 2013 [51]	Japan	N	32/32	66.8 ± 4.6/67.1 ± 2.8	NR	13.4 ± 1.9/13.2 ± 2.0
Lachman 2006 [39]	America	N	102/108	75.3 ± 7.4/74.6 ± 6.5	Total 77.6	14.3 ± 2.7/13.9 ± 3.1
Ferreira 2015 [30]	Brazil	N	22/22	66.2 ± 5.6/69.2 ± 4.8	68.2/86.3	12.9 ± 2.7/12.9 ± 2.5
Vaughn 2014 [52]	Astralia	N	25/23	69.0 ± 3.1/68.8 ± 3.5	NR	12.3 ± 2.9/12.7 ± 3.5
Fabre 2002 [31]	France	N	8/8	65.4 ± 2.2 /65.7 ± 1.5	87.5/75	11.2 ± 1.3/12.1 ± 1.4
Shan 2016 [44]	China	N	25/20	61.2 ± 5.3/59.1 ± 4.9	68/85	9.4 ± 3.1/8.8 ± 3.2
Li 2016 [45]	China	N	28/29	66.6 ± 4.0/65.9 ± 5.1	NR	>high school 50/48.3
Yang 2019 [46]	China	N	13/13	66.3 ± 4.3/65.9 ± 3.5	76.9/76.9	>high school 100/100
Hong 2017b [40]	Korea	N	13/12	75.9/73.2	83.3/46.1	NR
Hong 2017a [40]	Korea	MCI	10/12	77.9/75.9	70/75	NR
Bae 2019 [53]	Japan	MCI	41/42	75.5 ± 6.0/76.4 ± 5.0	43.9/52.4	11.1 ± 2.2/10.9 ± 2.3
Damirchi 2018a [54]	Iran	MCI	11/9	68.8 ± 3.7/69.1 ± 4.9	100/100	3.4 ± 1.0/3.2 ± 1.2
Damirchi 2018b [54]	Iran	MCI	13/9	67.8 ± 4.7/69.1 ± 4.9	100/100	2.8 ± 0.9/3.2 ± 1.2
Donnezan 2018 [28]	France	MCI	18/14	77.1 ± 1.44/79.2 ± 4	NR	6.1 ± 0.4/5.8 ± 0.4
Yoon 2018 [41]	Korea	MCI	20/23	73.8 ± 4.4/74.0 ± 4.3	70/69.6	8.1 ± 3.5/9.8 ± 4.4
Eggermont 2009 [32]	Korea	MCI	51/46	85.4	NR	NR
Lam 2010 [47]	China	MCI	135/194	77.2 ± 6.3/78.3 ± 6.6	NR	4.1 ± 4.3/2.6 ± 3.2
Scherder 2005 [33]	Netherlands	MCI	15/15	84 ± 6.4/86 ± 5.1	86.7/93.3	2.6 ± 1.1/2.7 ± 1.7
Sungkarat 2016 [48]	Thailand	MCI	33/33	68.3 ± 6.7/67.5 ± 7.3	93.9/78.8	11.4 ± 5.1/9.3 ± 5.5
Zhu 2018 [34]	China	MCI	29/31	70.3 ± 6.7/69 ± 7.3	51.7/67.7	>high school 86.2/90.3
Lü 2015 [42]	China	MCI	22/23	69 ± 3.83/70.43 ± 5.53	72.7/69.6	9.8 ± 2.8/9.5 ± 2.6
Nishiguchi 2015 [35]	Japan	N	24 /24	73.0 ± 4.8/73.5 ± 5.6	73.0 ± 4.8/73.5 ± 5.6	12.2 ± 2.2/13.0 ± 2.5

*N* normal cognition, *MCI* Mild Cognitive Impairment, *NR* No Reported, *E/C* Experiment group/Control group

As shown in Table 2, all or some of the exercise variables were reported in the 28 included studies. There were four types of physical exercise in the 28 included studies, as follows: AE (*n* = 9) [28–36], RE (*n* = 6) [37–42], MBE (*n* = 7) [18, 43–48], and MCE (*n* = 6) [49–54]. Exercise frequency varied from one to five times/week, with three times/week being the most common; exercise duration varied from 30 min to 90 min, with 60 min being most common; and exercise program length varied from 4 weeks to 52 weeks, with 24 weeks being most common. The index of exercise intensity varied between studies, but most studies adopted moderate-intensity exercise. Among the 28 studies, 15 included a passive control group and 13 included an active control group (social activities, *n* = 5; health education, *n* = 3; stretching exercises, *n* = 4; and cognitive training, *n* = 1).

**Table 2:** Intervention Characteristics Included in the Study

Study	Exercise type		Exercise prescription variables				Outcomes
	Experiment group	Control group	Length (week)	Frequency (times/week)	Session time (minites/times)	Intensity	
Brown 2009a [49]	MCE	Wait list	24	2	60	NR	(1)
Brown 2009b [49]	MCE	Wait list	24	2	60	NR	(1)
Eggenberger 2016 [28]	AE	Balance	8	3	30	NR	(6)
Gothe 2016 [62]	MBE	Stretching	8	3	NR	NR	(3), (5)
Albinet 2016 [29]	AE	Stretching	20	2	60	40–65%HRR	(3), (5), (7)
Kalbe 2018 [50]	MCE	Cognitive training	7	2	90	NR	(1)
Liu-ambrose 2010a [37]	RE	Balance	52	1	60	80–100%1RM	(5)
Liu-ambrose 2010b [37]	RE	Balance	52	2	60	80–100%1RM	(5)
Hariprasad 2013 [43]	MBE	Wait list	24	1	60	NR	(1), (7)
Norouzi 2019 [38]	RE	Social visit	4	3	60–80	NR	(3)
Nouchi 2013 [51]	MCE	Wait list	4	3	30	60–80%HR_max_	(1)
Lachman 2006 [39]	RE	Wait list	24	3	30	10RM	(1)
Ferreira 2015 [30]	AE	Social visit	24	3	40–50	60–80%HRR	(1)
Vaughn 2014 [52]	MCE	Wait list	16	2	60	NR	(8)
Fabre 2002 [31]	AE	Draw and sing	8	2	60	NR	(1)
Shan 2016 [44]	MBE	Wait list	12	5	60	NR	(1)
Li 2016 [45]	MBE	Health education	24	3	60	55–75%HR_max_	(1)
Yang 2019 [46]	MBE	Daily activities	8	3	45	NR	(3)
Hong 2017b [40]	RE	Wait list	12	2	60	15RM	(1)
Hong 2017a [40]	RE	Wait list	12	2	60	15RM	(1)
Bae 2019 [53]	MCE	Health education	24	2	90	NR	(4)
Damirchi 2018a [54]	MCE	Wait list	24	3	45	55–75%HRR, RPE13–15	(1)
Damirchi 2018b [54]	MCE	Wait list	24	3	45	55–75%HRR, RPE13–15	(1)
Donnezan 2018 [28]	AE	Wait list	12	2	60	60% HR_max_	(1)
Yoon 2018 [41]	RE	Stretching	16	3	60	RPE12–13	(1)
Eggermont 2009 [32]	AE	Social visit	6	5	30	NR	(1)
Lam 2010 [47]	MBE	Stretching	8	3	30	NR	(1)
Scherder 2005 [33]	AE	Wait list	6	3	30	NR	(1)
Sungkarat 2016 [48]	MBE	Health education	15	3	50	NR	(1)
Zhu 2018 [34]	AE	General care	12	3	35	60–80% HR_max_	(1)
Lü 2015 [42]	RE	Wait list	12	3	60	NR	(1)
Nishiguchi 2015 [35]	AE	Wait list	12	1	90	NR	(3)

Digit Span;(2) Sternberg task;(3)N-back task;(4) Corsi block-tapping task;(5) Verbal Span;(6) Executive Control task;(7) Spatial Span; (8)Letter-Number Sequencing test; *HRR* heart rate reserve, *HRmax* Maximum heart rate, *RPE* rating of perceived exertion, *VO2max* Maximum oxygen uptake, “*RM*” Repetition Maximum

### Methodological quality

The methodological quality of the included studies is reported in Table 3. The PEDro scores of the included studies ranged from 5 to 10 points, with an average of 7 points. The overall methodological quality was fair to excellent, with PEDro scores ≥6 for 15 studies (good), PEDro scores of 4–5 for 7 studies (fair), and PEDro scores of 9–10 for 6 studies (excellent). All the included studies carried out randomization, between-group comparisons, point measure, and measures of variability. A total of 16 studies used concealed allocation, 6 studies used blinding of assessors, blinding of subjects, and blindness of therapists, and 12 studies used an intent-to-treat analysis.

**Table 3:** Methodological Quality Assessment for Inclusion in the study

Study	Item1	Item2	Item3	Item4	Item5	Item6	Item7	Item8	Item9	Item10	Sum score
Brown 2009 [49]	1	0	1	0	0	0	1	0	1	1	5
Eggenberger 2016 [28]	1	1	1	1	1	1	1	0	1	1	9
Gothe 2016 [62]	1	1	1	1	1	1	1	0	1	1	9
Albinet 2016 [29]	1	0	1	0	0	0	1	0	1	1	5
Kalbe 2018 [50]	1	1	1	0	1	0	1	0	1	1	7
Liu-ambrose 2010 [37]	1	1	1	1	0	0	1	1	1	1	8
Hariprasad 2013 [43]	1	0	1	0	0	0	1	0	1	1	5
Norouzi 201 9[38]	1	0	1	0	0	0	1	0	1	1	5
Nouchi 2013 [51]	1	1	1	1	1	1	1	1	1	1	10
Lachman 2006 [39]	1	1	1	0	0	1	1	0	1	1	7
Ferreira 2015 [30]	1	0	1	0	0	1	1	1	1	1	7
Vaughn 2014 [52]	1	0	1	0	0	1	1	1	1	1	7
Fabre 2002 [31]	1	0	1	0	0	0	1	1	1	1	6
Shan 2016 [44]	1	1	1	1	1	1	1	0	1	1	9
Li 2016 [45]	1	1	1	1	1	1	1	1	1	1	10
Yang 2019 [46]	1	0	1	0	0	0	1	0	1	1	5
Hong 2017 [40]	1	0	1	0	0	0	1	1	1	1	6
Bae 2019 [53]	1	1	1	1	0	0	1	1	1	1	8
Damirchi 2018 [54]	1	0	1	0	0	0	1	0	1	1	5
Donnezan 2018 [28]	1	1	1	0	0	0	1	0	1	1	6
Yoon 2018 [41]	1	0	1	0	0	0	1	0	1	1	5
Eggermont 2009 [32]	1	1	1	0	1	0	1	1	1	1	8
Lam 2010 [47]	1	1	1	1	1	1	1	0	1	1	9
Scherder 2005 [33]	1	0	1	0	0	1	1	1	1	1	7
Sungkarat 2016 [48]	1	1	0	0	0	1	1	1	1	1	7
Zhu 2018 [34]	1	1	1	0	1	1	1	0	1	1	8
Lü 2015 [42]	1	1	1	0	0	1	1	0	1	1	7
Nishiguchi 2015 [35]	1	0	1	0	0	0	1	1	1	1	6

Item 1, randomization; Item 2, concealed allocation; Item 3, similar baseline; Item4, blinding of subjects; Item 5, blinding of therapists; Item 6, blinding of assessors; Item 7, more than 85% retention; Item 8, intent-to-treat analysis; Item 9, between-group comparison; Item 10, point measure and measures of variability; 1, explicitly described and present in details; 0, absent, inadequately described, or unclear

### Meta-analysis

**Fig. 2:**
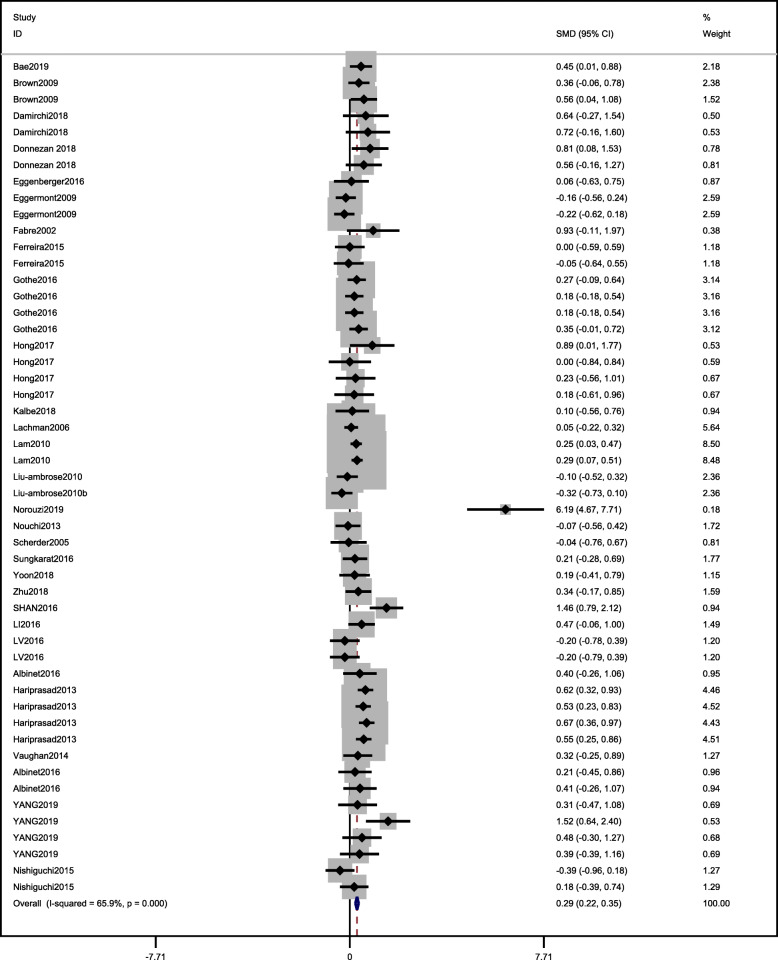
forest plot

**Fig. 3:**
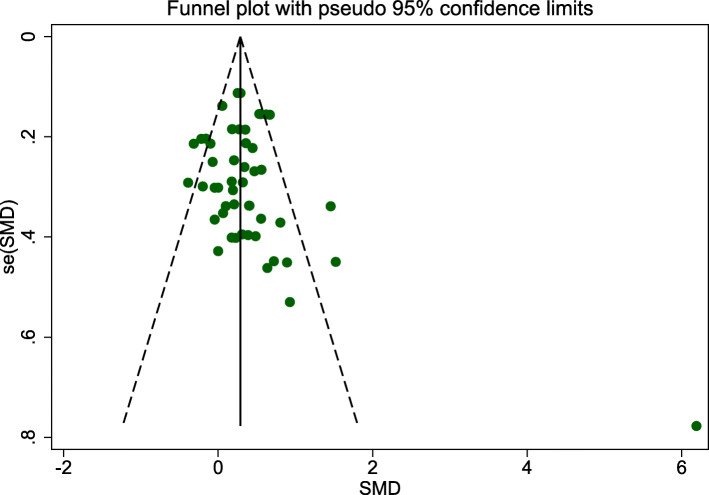
funnel plot

A total of 51 effects were included in the meta-analysis, and the overall ES was 0.29, *p* < 0.001, with a significant difference between the experimental and control groups. This indicates that exercise significantly improved WM in older adults. The heterogeneity test revealed a moderate degree of heterogeneity in the included studies (Table 4 and Fig. 2), so a random effect model was used to synthesize the data. The funnel plot in Fig. 3 was symmetrical, which indicates that there was no publication bias. Egger’s test showed that there was no publication bias in this study, which indicates that the small sample size in one of the included study did not affect the results (*t* = 1.46, *p* > | t | = 0.149 > 0.05; Table 5).

**Table 4:** Summary of Subgroup Analysis Results

Moderator	Grouping standard	*n (ES)*	Heterogeneity test results		The results of meta-analysis	
			*I*^*2*^	*P*	*ES,95%CI*	*P*
Measurement of WM testing	Digital Span Forward	13	86.1%	< 0.001	*SMD* = 0.38(0.24,0.51)	< 0.001
	Digital Span Backward	21	34.6%	0.061	*SMD =* 0.19(0.09,0.29)	< 0.001
	N-back (Accuracy)	6	7.4%	0.369	*SMD* = 0.21(0.002,0.41)	0.048
	N-back (Reaction Time)	3	68.6%	0.041	*SMD* = 0.55(0.14,0.95)	0.008
	Spatial Span	3	0%	0.735	*SMD* = 0.59(0.39,0.80)	< 0.001
	Miscellaneous	5	0%	0.978	*SMD* = 0.22(0.01,0.43)	0.038
Type	MCE	9	0%	0.639	*SMD =* 0.38(0.2,0.56)	< 0.001
	AE	13	0%	0.464	*SMD* = 0.04(−0.12,0.19)	0.66
	MBE	17	45.6%	0.21	*SMD =* 0.41(0.33,0.5)	< 0.001
	RE	12	86.0%	< 0.001	*SMD* = 0.06(−0.10,0.21)	0.50
Duration (minutes)	Short (≤45 min)	8	24.6%	0.233	*SMD* = 0.12(0.008,0.24)	0.037
	Moderate (45–60 min)	38	40.1%	0.006	*SMD* = 0.37(0.28,0.45)	< 0.001
	Long (> 60 min)	5	93.7%	< 0.001	SMD = 0.33(0.06,0.59)	< 0.017
Frequency (week/times)	Low (1–2times)	21	44.9%	0.014	*SMD* = 0.37(0.27,0.48)	< 0.001
	Moderate (3–4times)	27	69.0%	< 0.001	*SMD* = 0.25(0.16,0.34)	< 0.001
	High (≥5times)	3	90.0%	< 0.001	*SMD* = 0.06(−0.20,0.32)	0.628
Intervention period (weeks)	Short (≤12 weeks)	19	75.2%	< 0.001	*SMD =* 0.24(0.15,0.33)	< 0.001
	Moderate(12–24 weeks)	2	15.2%	0.261	*SMD =* 0.33(0.23,0.44)	< 0.001
	Long (> 24 weeks)	4	85.0%	< 0.001	*SMD =* 0.33(0.15,0.50)	< 0.001
Intensity	Low	29	54.8%	< 0.001	*SMD* = 0.32(0.25,0.40)	< 0.001
	Moderate	14	81.3%	< 0.001	*SMD* = 0.31(0.16,0.47)	< 0.001
	Vigorous	8	0%	0.577	*SMD = -*0.002(−0.20,0.19)	0.987
Control group	Active	28	70.6%	< 0.001	*SMD* = 0.22(0.14,0.30)	< 0.001
	Passive	23	54.9%	0.001	*SMD* = 0.38(0.28,0.48)	< 0.001
Cognitive status	MCI	17	27.5%	0.141	*SMD* = 0.22(0.14,0.30)	< 0.001
	Normal	32	73.5%	< 0.001	*SMD* = 0.30(0.23,0.36)	< 0.001
Age	> 76 yrs	18	51.9%	0.006	*SMD* = 0.33(0.24,0.42)	< 0.001
	≤75 yrs	33	70.7%	< 0.001	*SMD* = 0.24(0.14,0.33)	< 0.001

**Table 5:** Results of Egger’s Test

Std_EFF	Coef.	Std.Err.	*t*	*P > |t|*	95%*CI*
Slope	0.0991962	0.1395748	0.71	0.481	−0.1816588, 0.3800512
Bias	0.8738566	0.5967201	1.46	0.149	−0.3252973, 2.073011

### Subgroup analysis

#### WM measurements

The subgroup analysis revealed that the six WM measurements [55] significantly moderated the effect of exercise on WM (*Q*(5) = 16.63, *p* = 0.005). The ES of the SS results (Cohen’s *d* = 0.59) was greater than that of the n-back (Cohen’s *d* = 0.55), DSF (Cohen’s *d* = 0.38) and DSB results (Cohen’s *d* = 0.19).

#### Exercise prescription variables

The type of exercise intervention significantly moderated the effect of exercise on WM (*Q* (3) = 27.10, *p* < 0.001). The subgroup analysis revealed that the ES for older adults engaged in MBE (Cohen’s *d =* 0.41) was larger than for those engaged in MCE (Cohen’s *d =* 0.38). The ESs of AE (Cohen’s *d =* 0.04) and RE (Cohen’s *d =* 0.06) were not significantly different.

Exercise duration significantly moderated the effect of exercise on WM (*Q* (2) = 11.74, *p* < 0.003). The results of the subgroup analysis indicated that the ES for older adults engaged in a moderate-duration exercise (45–60 min) (Cohen’s *d* = 0.37) was larger than that for long-duration exercise (≥ 60 min) (Cohen’s *d =* 0.33) and short-duration exercise (≤ 45 min) (Cohen’s *d =* 0.12).

Exercise frequency significantly moderated the effect of exercise on WM (*Q* (2) = 8.29, *p* = 0.016). The subgroup analysis indicated that the ES for older adults engaged in a low-frequency exercise (1 or 2 times/week) (Cohen’s *d =* 0.37) was larger than that for those engaged in moderate-frequency exercise (3 or 4 times/week) (Cohen’s *d =* 0.25) or high-frequency exercise (≥ 5 times/week) (Cohen’s *d* = 0.06).

Exercise intensity significantly moderated the effect of exercise on WM (*Q* (2) = 9.39, *p* = 0.009). The subgroup analysis indicated that the ES for older adults engaged in a low-intensity exercise (Cohen’s *d =* 0.32) was larger than that for those engaged in moderate-intensity exercise (Cohen’s *d =* 0.31) or high-intensity exercise (Cohen’s *d* = − 0.002).

There were no significant differences in the ESs according to intervention period (*Q* (2) = 1.93, *p* = 0.381).

The active/passive control group significantly moderated the effect of exercise on WM (*Q* (1) = 5.85, *p* = 0.016).

#### Subject characteristics

There were no significant differences in the ESs according to cognitive status (*Q* (2) = 3.20, *p* = 0.074).

There were no significant differences in the ESs according to age (*Q* (1) = 2.07, *p* = 0.15].

## Discussion

### Overall analysis of exercise intervention effects

To the best of our knowledge, this is the first meta-analysis of RCTs investigating the effects of exercise prescription on WM. It is important to further our understanding on how exercise prescription could moderate the intervention effect. A previous meta-analysis revealed that regular physical exercise can improve WM in older adults [56], but included participants of all ages, from adolescents to older adults, and only 5 of the included studies were with older adults. The number of included studies in that meta-analysis was small, which limits the generalizability of those results. Additionally, no previous meta-analysis has investigated whether cognitive status influences the effect of exercise on WM in older adults with cognitive impairment.

The present meta-analysis included 28 studies and synthesized 51 ESs. The results further confirmed that exercise significantly improves WM in older adults, with a positive, significant small ES. Based on the results of this review, we believe that exercise is an effective way to improve WM in older adults, which is generally consistent with the results of previous meta-analyses [10, 23]. However, the current research found a moderate heterogeneity between the included studies, which may be caused by factors such as different WM measurement tools, the cognitive status of older adults, and the specific features of physical exercise.

### Subgroup analysis of exercise intervention effects

#### WM measurements

This study found that the intervention effect of physical exercise on the WM of older adults was moderated by the WM measurement tools. WM comprises many sub-components, such as encoding, maintaining, and manipulating information [57]. Different measurement tools differ in their investigation of these different WM subcomponents. For example, the DSF and verbal span tasks mainly assess retention in WM. The n-back and DSB tasks not only assess memory retention, but also the manipulation of WM. The tools used to assess WM are diverse, and can be divided into two categories – task span and n-back tasks [58]. In the included studies, WM was mostly tested using the DST, because the DSF task does not involve additional manipulations of the memory content. The DSB task not only assesses retention, but also manipulation. By comparing the differences between the two tasks, the intervention effect of a single component can be determined.

The current study found that the intervention effect of physical exercise on the DSF task is better than that on the DSB task, which is similar to previous results [59]. This shows that the intervention effect of physical exercise on relatively simple WM is better, but the intervention effect on task manipulation is poor, which may because the scoring method of the DSF is not very sensitive and cannot reflect the changes in WM. The use of a more accurate digit-letter sequence task could be explored in this context [60].

The n-back task is arguably the most commonly used continuous updating test and shows acceptable convergence with conceptually distinct measures of WM, including complex span and serial reordering tasks [61]. This task can be classified as 1-back, 2-back, and 3-back. The subjects respond according to whether the current information is the same as the previous information. This study found significant ESs of the n-back reaction time and accuracy. However, few studies on this were included, so this explanation should be treated with some caution. Furthermore, the difficulty of n-back task could also affect the intervention effect, and the intervention effect on the accuracy of the relatively simple 1-back task is not as good as that on 2-back accuracy [46, 62].

#### Exercise prescription variables

The current meta-analysis also evaluated the effects of exercise prescription on the exercise effects on WM. The present results revealed that the type of physical exercise is a potential regulatory variable in this relationship.

#### The moderating effect of exercise type

Our findings indicate that exercise type moderates the influence of exercise on WM. Exercise type is an important feature of physical exercise, and most of the earlier studies adopted an AE intervention [63]. The intervention effects of RE [64], MCE [65], and MBE [66] have also been confirmed. However, it is worth noting that the present results revealed no significant intervention effects of AE and RE on WM, but did find significant effects of MCE and MBE. Previous meta-analyses also reported that AE and RE do not improve WM in older adults [55, 67, 68], but, when combined, they become effective in improving WM [69]. As the most commonly used method of physical exercise intervention, many studies have shown that AE and RE cause changes in brain function [70, 71] and can improve cognitive functioning [72, 73]. The inconsistency in the results of previous studies may due to the multi-component nature of WM, the various WM measurement tools, and large individual differences in cognitive functioning.

Compared with a single form of exercise, MCE and MBE are relatively complex, in that they involve multi-point memory and adopt characteristics of aerobic, resistance, balance, and stretching movements. The effects of various forms of exercise may produce complementary neurobiological and physiological effects on WM, especially when the form of exercise engages similar systems to those engaged in WM tasks. Tai Chi Chuan perfectly integrates traditional philosophy, the theory of traditional Chinese medicine, and the five-element theory; it also combines physical movement with respiration, mind with consciousness, consciousness with the body, and qi with the body. It strives to achieve a unity of mind, consciousness, strength, qi(a Chinese concept of energy), and shape, while constantly adjusting the direction, range, power, and speed of movement. This practice requires not only memory, but also a variety of higher-level cognitive functions to maintain postural stability. It has also been reported to improve brain structure and cognitive function by improving cardiovascular function and coordination ability [74]. The amplitude and latency of event-related potentials in older adults who have been practicing Tai Chi Chuan for a long time have been reported to significantly change [75]. Several previous experimental studies [10, 74] and meta-analyses [23, 67, 76, 77] have shown that MCE and MBE may have a greater positive impact on the cognitive function of older adults than other types of exercise.

#### The moderating effect of exercise frequency

The subgroup analysis indicated that exercise frequency moderates the influence of exercise on WM. Low- and moderate-frequency exercise had a positive exercise effect on WM in older adults, while high-frequency exercise had no such positive effect. The results of a previous meta-analysis indicated that both high-frequency and low-frequency physical exercise can improve cognitive functioning in older adults [78]. The difference between these previous results and those of the current study may be related to the lack of literature on an exercise frequency of 5 times or more per week. Our result may not represent a true effect, and the results should be interpreted with caution, and more research is needed.

#### The moderating effect of exercise intensity

The subgroup analysis indicated that exercise intensity moderates the influence of exercise on WM. There was heterogeneity in the intervention effect between different exercise intensities. Moderate- and low-intensity exercise was found to effectively improve WM in older adults, while high-intensity physical exercise had no intervention effect. Several meta-analyses have made the same conclusions [79, 80]. It has been agreed that moderate-intensity physical exercise can effectively improve WM in older adults, which is also in line with the exercise intensity advocated by the American Sports Medical Association and the World Health Organization.

Physical exercise can result in structural brain changes, such as increased hippocampal volume [12] and gray matter volume [66]. Many studies have found that exercise intensity plays an important role in improving cognitive performance [81, 82]. A recent meta-analysis found that both high-intensity and low-intensity physical exercise improved executive functioning, with no significant differences between the two intensities [83]. However, only 2 of the included studies adopted high-intensity physical exercise. Thus, this result should be interpreted with caution, and more studies are needed.

#### The moderating effect of exercise duration

The subgroup analysis indicated that exercise duration moderates the influence of exercise on WM, whereby the effect of exercise tends to increase with a longer duration. Most researchers have implemented sessions that last 30–60 min; however, some research has failed to clearly state the exercise duration or to distinguish between the warm-up, main exercise, and cool-down of each session. Many studies have suggested that 20 min of physical exercise can significantly improve cognitive functioning in older adults [72, 84]. Exercise durations that are too short are insufficient to induce changes in body arousal level, brain structure, and function. However, exercise sessions that are too long may cause excessive fatigue in older adults, and does not induce brain plasticity. Therefore, it is important to define the duration that will most effectively induce such changes [68]. Future studies should thus clarify the intervention effect according to exercise duration.

#### The moderating effect of intervention period

The subgroup analysis indicated that the intervention period does not moderate the effect of exercise on WM. The short, medium, and long intervention periods could all improve WM in older adults. The current findings replicate several earlier studies. For example, one study reported no relationship between exercise effect and intervention period [79], but some studies have proposed that the effect of a long intervention period [85] or short intervention period [86] is better.

The most commonly used intervention periods in the included studies were 8 weeks, 12 weeks, and 24 weeks; those more than 24 weeks were rare, so these results should be interpretated with caution. While a long intervention period may improve cognitive performance, the cognitive performance of older adults may decline over time, thus offsetting the effect of the intervention. Future studies need to prolong the length of exercise and increase the number of follow-ups to evaluate whether cognitive status differences between intervention and control groups increase with age.

#### Subject characteristics

The subgroup analysis indicated that the cognitive status of subjects did not moderate the influence of exercise on WM. However, this study found that a larger effect of the intervention in older adults with normal cognition than in older adults with MCI. As a possible moderating variable, many researchers have examined the role of cognitive status in the effect of interventions on cognition. While research has revealed significant intervention-related improvements in healthy older adults, older patients with MCI, and even patients with dementia, these results have not been consistent in the cognitive domain or in the magnitudes of improvement [12, 78, 87]. The discrepancy of these results may be caused by the small number of included studies.

The subgroup analysis indicated that age does not moderate the effect of exercise on WM. These results are not consistent with those of Colcombe and Kramer, who found that physical exercise has the greatest impact on cognitive function in adults aged 66–70 years, followed by those in the 71–80 years bracket, and has the least impact on the cognitive function of adults aged 55–65 years [10]. The reason for this inconsistency may be different in the measurement tools of cognitive sub-domain.

### Strengths and limitations

This study’s primary strength was the exclusive inclusion of RCTs. In previous studies, the inclusion of cross-sectional studies have introduced confounding variables that affect the authenticity of the research results. Another strength of this study is that it analyzed the moderating effect of exercise prescription features. These results thus provide a theoretical basis for identifying optimal exercise prescription parameters.

This meta-analysis also has several limitations that should be overcome in future research. First, while there is a variety of measurement tools to assess WM, this study mainly used DST results as the primary outcome. Second, the included studies have some methodological flaws, such as the absence of blinding. Third, there are no standards for exercise intensity and exercise duration in the literature, which makes it difficult to determine the most effective intervention parameters.

## Conclusion

This systematic meta-analytic review indicates that exercise is a promising way to improve WM in older adults. We found that the best physical exercise prescription for improving WM in older adults is moderate intensity MBE or MCE sessions of 45–60 min performed 3–4 times a week, for at least 12 weeks. The effect of the intervention was not affected by age or cognitive status. However, due to the limited inclusion of studies, the optimal exercise prescription needs to be confirmed in future work.

## Supplementary Information

**Additional file 1.**

## Data Availability

All the data is in the supplementary data file.

## Abbreviations

AE: Aerobic exercise
DST: Digit Span Test
DSF: Digit Span Test-Forward
DSB: Digit Span Test-Backward
ES: Effect size
EC: Executive control
M: Mean
MBE: Mind–body exercise
MCI: Mild cognitive impairment
MCE: Multi-component exercise
PEDro: Physiotherapy Evidence Database
PFC: Prefrontal cortex
RE: Resistance exercise
RCT: Randomized controlled trial
SS: Spatial span
SD: Standard deviation
WM: Working memory
