# Supplementary material for: Effects of physical exercise on working memory in older adults: a systematic and meta-analytic review
Source: Eur Rev Aging Phys Act. 2021 Sep 17;18:18. doi: 10.1186/s11556-021-00272-y (PMC8447686; doi:10.1186/s11556-021-00272-y)
Supplement: Supplementary file 1 — Additional file 1. [file 11556_2021_272_MOESM1_ESM.zip › 11556_2021_272_MOESM1_ESM/Supplementary Material.docx]

**Included studies characteristics**

| Studies | exp_n | exp_m | exp_sd | cont_n | cont_m | cont_sd | cognitive status | outcomes | type | length | frequency | session time | intensity | age |
| --- | --- | --- | --- | --- | --- | --- | --- | --- | --- | --- | --- | --- | --- | --- |
| Bae2019 | 41 | 6.87 | 1.27 | 42 | 6.32 | 1.2 | MCI | DSB | multi-component | 24 | 2 | 90 | NO repert | ＞75 |
| Brown2009 | 66 | 6.2 | 2.1 | 34 | 5.5 | 1.6 | N | DSB | multi-component | 24 | 2 | 60 | NO repert | ＞75 |
| Brown2009 | 26 | 6.5 | 2 | 34 | 5.5 | 1.6 | N | DSB | multi-component | 24 | 2 | 60 | NO repert | ＜75 |
| Damirchi2018 | 11 | 7.81 | 2.08 | 9 | 6.44 | 2.24 | MCI | DSB | multi-component | 24 | 3 | 45 | 55-75%HRR,RPE13-15 | ＜75 |
| Damirchi2018 | 13 | 8.37 | 2.93 | 9 | 6.44 | 2.24 | MCI | DSF | multi-component | 24 | 3 | 45 | 55-75%HRR,RPE13-15 | ＜75 |
| Combourieu 2018 | 18 | 4.7 | 1.2 | 14 | 3.8 | 1 | MCI | DSB | multi-component | 24 | 3 | 45 | 55-75%HRR,RPE13-15 | ＞75 |
| Combourieu 2018 | 18 | 5.9 | 0.9 | 14 | 5.4 | 0.9 | MCI | DSF | multi-component | 24 | 3 | 45 | 55-75%HRR,RPE13-15 | ＞75 |
| Eggenberger2016 | 19 | 10.84 | 4.8 | 14 | 10.57 | 3.3 | N | executive control | aerobic | 8 | 3 | 30 | moderare-high | ＜75 |
| Eggermont2009 | 51 | 3.69 | 1.28 | 46 | 3.89 | 1.25 | MCI | DSB | aerobic | 6 | 5 | 30 | NO repert | ＞75 |
| Eggermont2009 | 51 | 4.63 | 1.64 | 46 | 4.98 | 1.57 | MCI | DSF | aerobic | 6 | 5 | 30 | NO repert | ＞75 |
| Fabre2002 | 8 | 6.1 | 0.7 | 8 | 5.6 | 0.3 | N | DSF | aerobic | 8 | 2 | 60 | NO repert | ＜75 |
| Ferreira2015 | 22 | 4.1 | 0.9 | 22 | 4.1 | 0.9 | N | DSB | aerobic | 24 | 3 | 40-50 | 60-80%HRR | ＜75 |
| Ferreira2015 | 22 | 4.9 | 1.13 | 22 | 5 | 2.93 | N | DSF | aerobic | 24 | 3 | 40-51 | 60-81%HRR | ＜75 |
| Gothe2016 | 61 | 31.64 | 8.71 | 57 | 29.3 | 8.32 | N | word span(total) | mind-body | 8 | 3 | NO repert | NO repert | ＜75 |
| Gothe2016 | 61 | 17.97 | 6.68 | 57 | 16.76 | 6.81 | N | word span(partial) | mind-body | 8 | 3 | NO repert | NO repert | ＜75 |
| Gothe2016 | 61 | 0.98 | 0.05 | 57 | 0.97 | 0.06 | N | 1-back（accuracy） | mind-body | 8 | 3 | NO repert | NO repert | ＜75 |
| Gothe2016 | 61 | 0.87 | 0.11 | 57 | 0.82 | 0.17 | N | 2-back（accuracy） | mind-body | 8 | 3 | NO repert | NO repert | ＜75 |
| Hong2017 | 10 | 2.17 | 1.52 | 12 | 1.08 | 0.91 | MCI | DSB | resistance | 12 | 2 | 60 | moderate | ＞75 |
| Hong2017 | 10 | 4 | 0.81 | 12 | 4 | 1.34 | MCI | DSF | resistance | 12 | 2 | 60 | moderate | ＞75 |
| Hong2017 | 12 | 3.42 | 1.5 | 13 | 3.15 | 0.8 | N | DSB | resistance | 12 | 2 | 60 | moderate | ＞75 |
| Hong2017 | 12 | 5.75 | 1.21 | 13 | 5.54 | 1.19 | N | DSF | resistance | 12 | 2 | 60 | moderate | ＞75 |
| Kalbe2018 | 18 | 8.06 | 2.58 | 17 | 7.82 | 2.27 | N | DSB | multi-component | 7 | 2 | 90 | moderate | ＜75 |
| Lachman2006 | 102 | 5.22 | 1.44 | 108 | 5.14 | 1.53 | N | DSB | resistance | 24 | 3 | 30 | 10RM | ＞75 |
| Lam2011 | 135 | 2.6 | 1.3 | 194 | 2.3 | 1.1 | MCI | DSB | mind-body | 8 | 3 | 30 | moderate | ＞75 |
| Lam2011 | 135 | 6.9 | 1.2 | 194 | 6.5 | 1.5 | MCI | DSF | mind-body | 8 | 3 | 30 | moderate | ＞75 |
| Liu-ambrose2010 | 46 | 3.8 | 2.1 | 42 | 4 | 1.9 | N | DSB | resistance | 52 | 1 | 60 | 80-100%1RM | ＜75 |
| Liu-ambrose2010b | 47 | 3.4 | 1.9 | 42 | 4 | 1.9 | N | DSB | resistance | 52 | 2 | 60 | 80-100%1RM | ＜75 |
| Norouzi2019 | 20 | 7.72 | 0.11 | 20 | 6.87 | 0.16 | N | DSF | resistance | 4 | 3 | 60-80 | moderate | ＜75 |
| Nouchi2014 | 32 | 4.6 | 1.58 | 32 | 4.71 | 1.51 | N | DSB | multi-component | 4 | 3 | 30 | 60-80%HR_max_ | ＜75 |
| Scherder2005 | 15 | 10.8 | 3.1 | 15 | 10.93 | 2.69 | MCI | DSB | aerobic | 6 | 3 | 30 | moderate | ＞75 |
| Sungkarat2016 | 33 | 13.8 | 3.2 | 33 | 13.2 | 2.6 | MCI | DSB | mind-body | 15 | 3 | 50 | moderate | ＜75 |
| Yoon2018 | 20 | 10.7 | 1.34 | 23 | 10.39 | 1.83 | MCI | DSB | resistance | 16 | 3 | 60 | RPE12-13 | ＜75 |
| Zhu2018 | 29 | 16.8 | 2.2 | 31 | 15.9 | 3 | MCI | DSB | aerobic | 12 | 3 | 35 | 60-80% HR_max_ | ＜75 |
| Shan2016 | 25 | 11.8 | 3.15 | 20 | 7.57 | 2.56 | N | DSF | mind-body | 12 | 5 | 60 | moderate | ＜75 |
| Li2016 | 28 | 8.72 | 3.19 | 29 | 7.3 | 2.86 | N | DSF | mind-body | 24 | 3 | 60 | 55-75%HR_max_ | ＜75 |
| Lü2016 | 22 | 3.8 | 0.9 | 23 | 4 | 1.1 | MCI | DSB | resistance | 12 | 3 | 60 | moderate | ＜75 |
| Lü2016 | 22 | 7.4 | 1.4 | 23 | 7.7 | 1.6 | MCI | DSF | resistance | 12 | 3 | 60 | moderate | ＜75 |
| Albinet2016 | 19 | 26.2 | 1.8 | 17 | 25.4 | 2.2 | N | 2-back(accuracy) | aerobic | 20 | 2 | 60 | moderate | ＜75 |
| Hariprasad2013 | 87 | 7.77 | 1.31 | 87 | 6.84 | 1.66 | N | DSF | mind-body | 24 | 1 | 60 | low | ＞75 |
| Hariprasad2013 | 87 | 5.3 | 1.23 | 87 | 4.51 | 1.7 | N | DSB | mind-body | 24 | 1 | 61 | low | ＞75 |
| Hariprasad2013 | 87 | 7.8 | 1.55 | 87 | 6.77 | 1.53 | N | spitial span | mind-body | 25 | 1 | 62 | low | ＞75 |
| Hariprasad2013 | 87 | 5.68 | 1.87 | 87 | 4.65 | 1.86 | N | spitial span | mind-body | 26 | 1 | 63 | low | ＞75 |
| Vaughan2014 | 25 | 16.3 | 3.8 | 23 | 15.2 | 3 | N | digit letter sequence | multi-component | 16 | 2 | 60 | NO repert | ＜75 |
| Albinet2016 | 19 | 26.6 | 7.7 | 17 | 25.4 | 2.2 | N | word span | aerobic | 20 | 2 | 60 | moderare-high | ＜75 |
| Albinet2016 | 19 | 37.8 | 7.8 | 17 | 34.3 | 9.5 | N | spitial span | aerobic | 20 | 2 | 60 | moderare-high | ＜75 |
| Yang2019 | 13 | 0.74 | 0.96 | 13 | 0.53 | 0.07 | N | 1-back（accuracy） | mind-body | 8 | 3 | 45 | NO repert | ＜75 |
| Yang2019 | 13 | -315.8 | 54.1 | 13 | -405 | 62.8 | N | 1-back（reaction time） | mind-body | 8 | 3 | 45 | NO repert | ＜75 |
| Yang 2019 | 13 | -373.3 | 110.6 | 13 | -417.7 | 67.4 | N | 2-back（reaction time） | mind-body | 8 | 3 | 45 | NO repert | ＜75 |
| Yang 2019 | 13 | 0.64 | 0.11 | 13 | 0.4 | 0.87 | N | 2-back(accuracy) | mind-body | 8 | 3 | 45 | NO repert | ＜75 |
| Nishiguchi2015 | 24 | 94.3 | 4.8 | 24 | 95.9 | 3.3 | N | 1-back（accuracy） | aerobic | 12 | 1 | 90 | NO repert | ＜75 |
| Nishiguchi2015 | 24 | -1118 | 184 | 24 | -1148 | 157 | N | 1-back（reaction time） | aerobic | 12 | 1 | 90 | NO repert | ＜75 |

Note：exp_n, sample size of experimental group; exp_m, mean value of experimental group; exp_sd, standard deviation of experimental group; cont_n, sample size of control group; cont_m, mean value of control group; cont_sd, standard deviation of control group; DSF, digit span forward; DSB, digit span backward;
