# Supplementary material for: Effects of physical exercise on working memory in older adults: a systematic and meta-analytic review
Source: Eur Rev Aging Phys Act. 2021 Sep 17;18:18. doi: 10.1186/s11556-021-00272-y (PMC8447686; doi:10.1186/s11556-021-00272-y)
Supplement: Supplementary file 1 — Additional file 1. [file 11556_2021_272_MOESM1_ESM.zip › 11556_2021_272_MOESM1_ESM/Supplementary Material.pdf]

Included studies characteristics

Studies	exp_n	exp_m	exp_sd	cont_n	cont_m	cont_sd	cognitive status	outcomes	type	length	frequency	session time	intensity	age
Bae2019	41	6.87	1.27	42	6.32	1.2	MCI	DSB	multi-component	24	2	90	NO repert	＞75
Brown2009	66	6.2	2.1	34	5.5	1.6	N	DSB	multi-component	24	2	60	NO repert	＞75
Brown2009	26	6.5	2	34	5.5	1.6	N	DSB	multi-component	24	2	60	NO repert	＜75
Damirchi2018	11	7.81	2.08	9	6.44	2.24	MCI	DSB	multi-component	24	3	45	55-75%HRR,RPE13-15	＜75
Damirchi2018	13	8.37	2.93	9	6.44	2.24	MCI	DSF	multi-component	24	3	45	55-75%HRR,RPE13-15	＜75
Donnezan 2018	18	4.7	1.2	14	3.8	1	MCI	DSB	multi-component	24	3	45	55-75%HRR,RPE13-15	＞75
Donnezan 2018	18	5.9	0.9	14	5.4	0.9	MCI	DSF	multi-component	24	3	45	55-75%HRR,RPE13-15	＞75
Eggenberger2016	19	10.84	4.8	14	10.57	3.3	N	executive control	aerobic	8	3	30	moderare-high	＜75
Eggermont2009	51	3.69	1.28	46	3.89	1.25	MCI	DSB	aerobic	6	5	30	NO repert	＞75

Eggermont2009	51	4.63	1.64	46	4.98	1.57	MCI	DSF	aerobic	6	5	30	NO repert	＞75
Fabre2002	8	6.1	0.7	8	5.6	0.3	N	DSF	aerobic	8	2	60	NO repert	＜75
Ferreira2015	22	4.1	0.9	22	4.1	0.9	N	DSB	aerobic	24	3	40-50	60-80%HRR	＜75
Ferreira2015	22	4.9	1.13	22	5	2.93	N	DSF	aerobic	24	3	40-51	60-81%HRR	＜75
Gothe2016	61	31.64	8.71	57	29.3	8.32	N	word span(total)	mind-body	8	3	NO repert	NO repert	＜75
Gothe2016	61	17.97	6.68	57	16.76	6.81	N	word span(partial)	mind-body	8	3	NO repert	NO repert	＜75
Gothe2016	61	0.98	0.05	57	0.97	0.06	N	1-back（accuracy）	mind-body	8	3	NO repert	NO repert	＜75
Gothe2016	61	0.87	0.11	57	0.82	0.17	N	2-back（accuracy）	mind-body	8	3	NO repert	NO repert	＜75
Hong2017	10	2.17	1.52	12	1.08	0.91	MCI	DSB	resistance	12	2	60	moderate	＞75
Hong2017	10	4	0.81	12	4	1.34	MCI	DSF	resistance	12	2	60	moderate	＞75
Hong2017	12	3.42	1.5	13	3.15	0.8	N	DSB	resistance	12	2	60	moderate	＞75
Hong2017	12	5.75	1.21	13	5.54	1.19	N	DSF	resistance	12	2	60	moderate	＞75
Kalbe2018	18	8.06	2.58	17	7.82	2.27	N	DSB	multi-component	7	2	90	moderate	＜75
Lachman2006	102	5.22	1.44	108	5.14	1.53	N	DSB	resistance	24	3	30	10RM	＞75
Lam2010	135	2.6	1.3	194	2.3	1.1	MCI	DSB	mind-body	8	3	30	moderate	＞75
Lam2010	135	6.9	1.2	194	6.5	1.5	MCI	DSF	mind-body	8	3	30	moderate	＞75
Liu-ambrose2010	46	3.8	2.1	42	4	1.9	N	DSB	resistance	52	1	60	80-100%1RM	＜75

Liu-ambrose2010b	47	3.4	1.9	42	4	1.9	N	DSB	resistance	52	2	60	80-100%1RM	＜75
Norouzi2019	20	7.72	0.11	20	6.87	0.16	N	DSF	resistance	4	3	60-80	moderate	＜75
Nouchi2013	32	4.6	1.58	32	4.71	1.51	N	DSB	multi-component	4	3	30	60-80%HR_{max}	＜75
Scherder2005	15	10.8	3.1	15	10.93	2.69	MCI	DSB	aerobic	6	3	30	moderate	＞75
Sungkarat2016	33	13.8	3.2	33	13.2	2.6	MCI	DSB	mind-body	15	3	50	moderate	＜75
Yoon2018	20	10.7	1.34	23	10.39	1.83	MCI	DSB	resistance	16	3	60	RPE12-13	＜75
Zhu2018	29	16.8	2.2	31	15.9	3	MCI	DSB	aerobic	12	3	35	60-80% HR_{max}	＜75
SHAN2016	25	11.8	3.15	20	7.57	2.56	N	DSF	mind-body	12	5	60	moderate	＜75
LI2016	28	8.72	3.19	29	7.3	2.86	N	DSF	mind-body	24	3	60	55-75%HR_{max}	＜75
LV2016	22	3.8	0.9	23	4	1.1	MCI	DSB	resistance	12	3	60	moderate	＜75
LV2016	22	7.4	1.4	23	7.7	1.6	MCI	DSF	resistance	12	3	60	moderate	＜75
Albinet2016	19	26.2	1.8	17	25.4	2.2	N	2-back(accuracy)	aerobic	20	2	60	moderate	＜75
Hariprasad2013	87	7.77	1.31	87	6.84	1.66	N	DSF	mind-body	24	1	60	low	＞75
Hariprasad2013	87	5.3	1.23	87	4.51	1.7	N	DSB	mind-body	24	1	61	low	＞75
Hariprasad2013	87	7.8	1.55	87	6.77	1.53	N	spitial span	mind-body	25	1	62	low	＞75

Hariprasad2013	87	5.68	1.87	87	4.65	1.86	N	spitial span	mind-body	26	1	63	low	＞75
Vaughan2014	25	16.3	3.8	23	15.2	3	N	digit letter sequence	multi-component	16	2	60	NO repert	＜75
Albinet2016	19	26.6	7.7	17	25.4	2.2	N	word span	aerobic	20	2	60	moderare-high	＜75
Albinet2016	19	37.8	7.8	17	34.3	9.5	N	spitial span	aerobic	20	2	60	moderare-high	＜75
YANG2019	13	0.74	0.96	13	0.53	0.07	N	1-back（accuracy）	mind-body	8	3	45	NO repert	＜75
YANG2019	13	-315.8	54.1	13	-405	62.8	N	1-back（reaction time）	mind-body	8	3	45	NO repert	＜75
YANG2019	13	-373.3	110.6	13	-417.7	67.4	N	2-back（reaction time）	mind-body	8	3	45	NO repert	＜75
YANG2019	13	0.64	0.11	13	0.4	0.87	N	2-back(accuracy)	mind-body	8	3	45	NO repert	＜75
Nishiguchi2015	24	94.3	4.8	24	95.9	3.3	N	1-back（accuracy）	aerobic	12	1	90	NO repert	＜75
Nishiguchi2015	24	-1118	184	24	-1148	157	N	1-back（reaction time）	aerobic	12	1	90	NO repert	＜75

Note：exp_n, sample size of experimental group; exp_m, mean value of experimental group; exp_sd, standard deviation of experimental group; cont_n, sample size of control group; cont_m, mean value of control group; cont_sd, standard deviation of control group; DSF, digit span forward; DSB, digit span backward;
