# Supplementary figures and images for: Effects of physical exercise on working memory in older adults: a systematic and meta-analytic review
Source: Eur Rev Aging Phys Act. 2021 Sep 17;18:18. doi: 10.1186/s11556-021-00272-y (PMC8447686; doi:10.1186/s11556-021-00272-y)

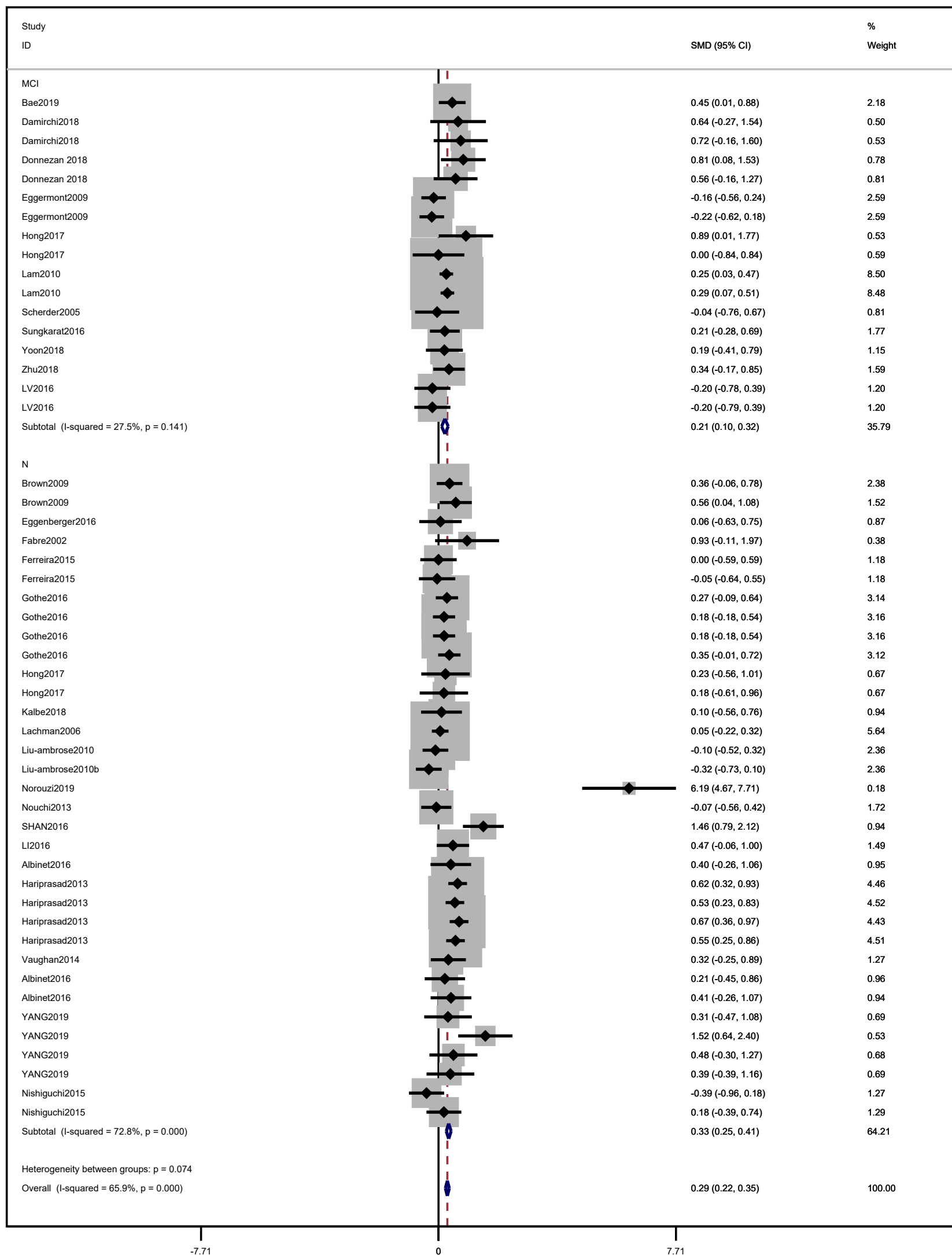

Supplement: Supplementary file 1 — Additional file 1. [file 11556_2021_272_MOESM1_ESM.zip › 11556_2021_272_MOESM1_ESM/subgroup - cognitive status.pdf]

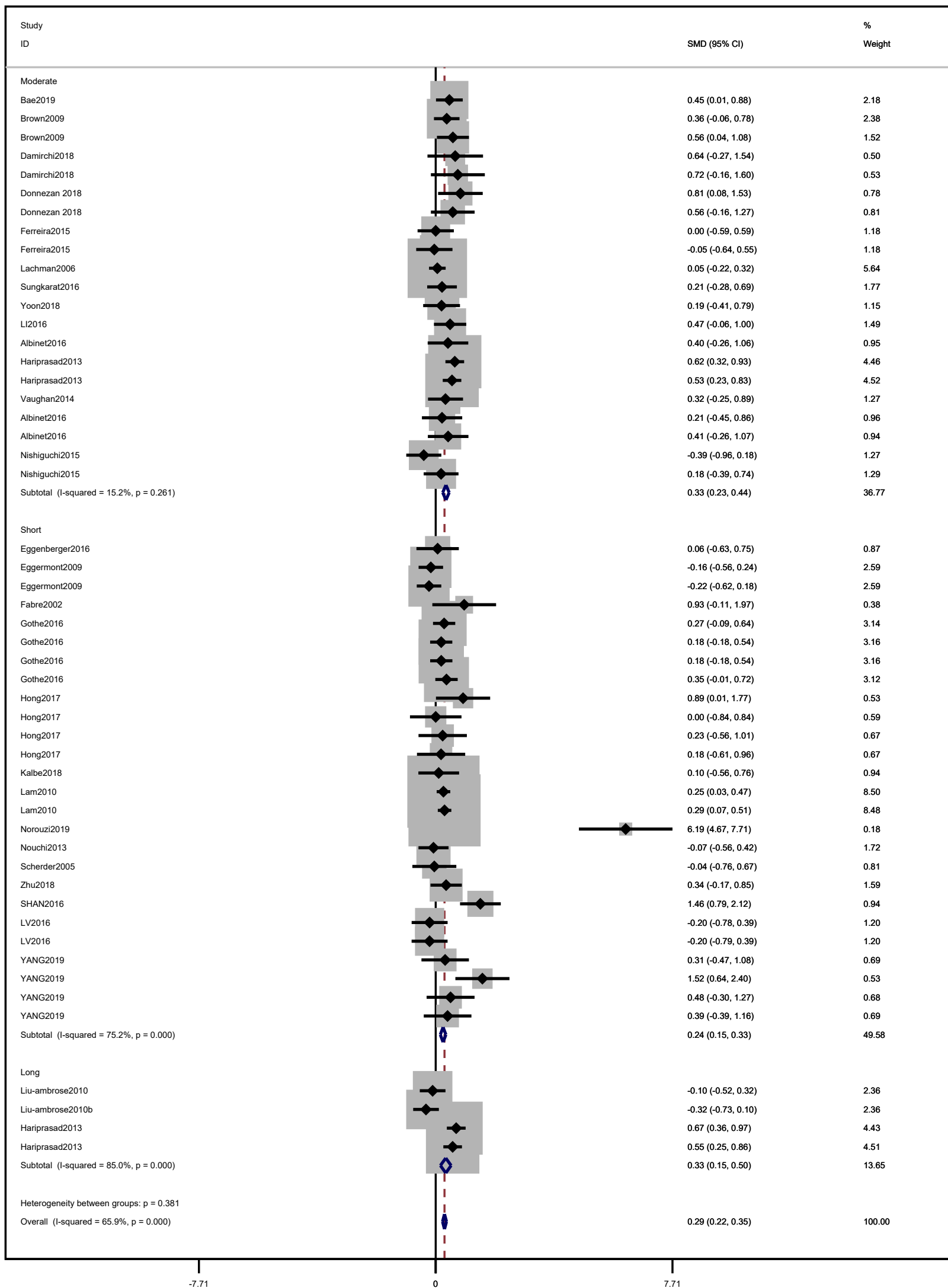

subgroup forest plot - intervention group

Supplement: Supplementary file 1 — Additional file 1. [file 11556_2021_272_MOESM1_ESM.zip › 11556_2021_272_MOESM1_ESM/subgroup- intervention group.pdf]

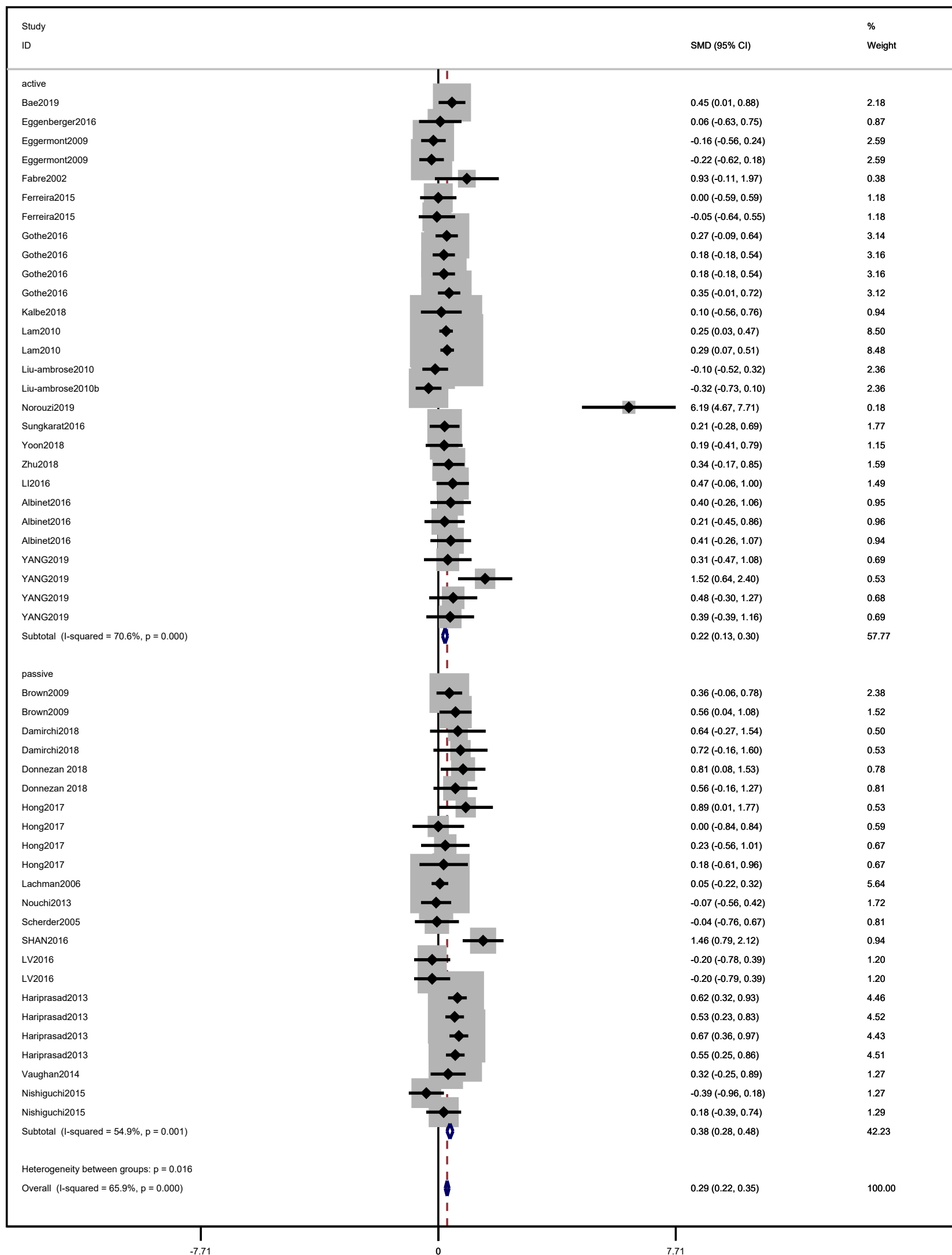

Supplement: Supplementary file 1 — Additional file 1. [file 11556_2021_272_MOESM1_ESM.zip › 11556_2021_272_MOESM1_ESM/subgroup-active-passive control group.pdf]

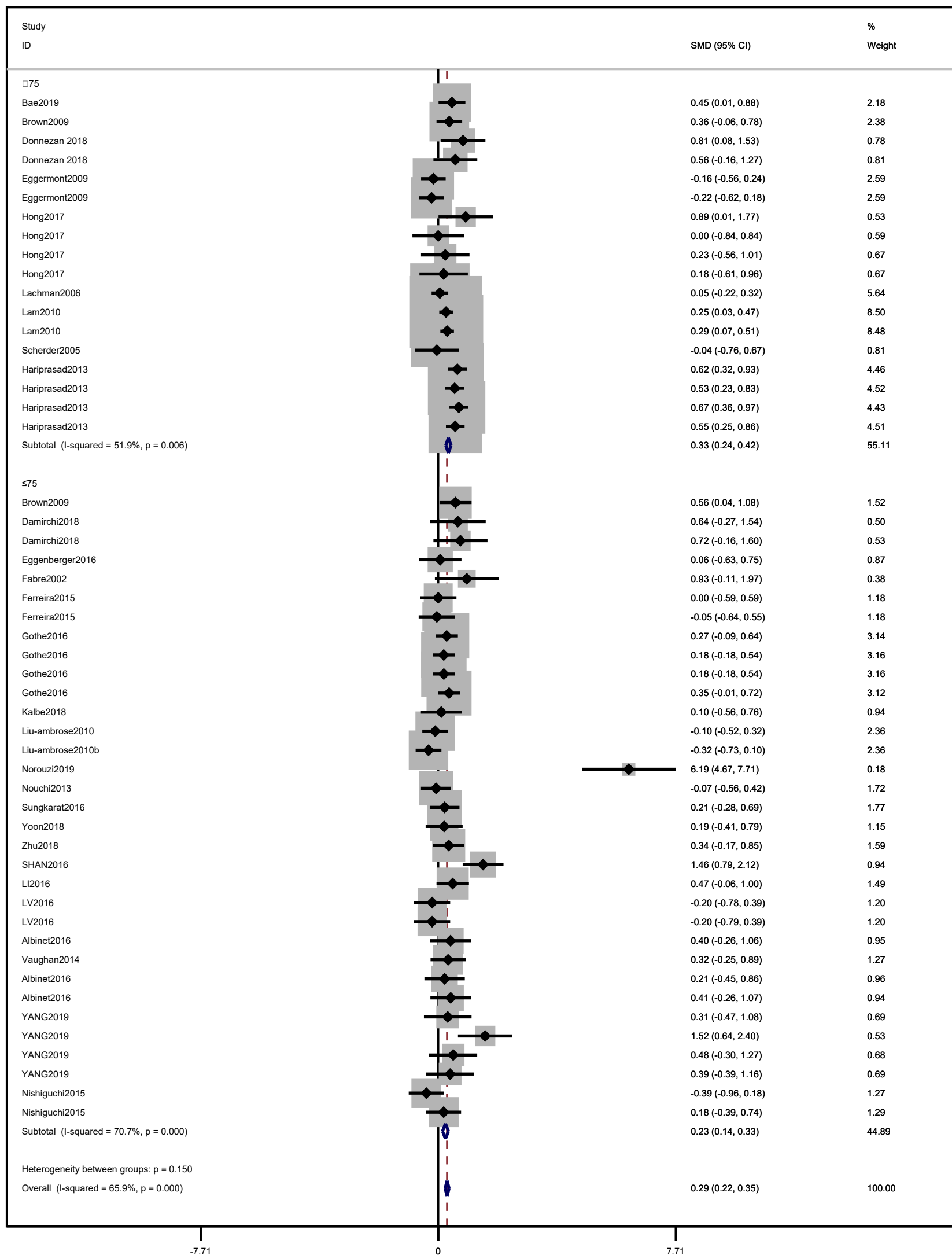

Supplement: Supplementary file 1 — Additional file 1. [file 11556_2021_272_MOESM1_ESM.zip › 11556_2021_272_MOESM1_ESM/subgroup-age.pdf]

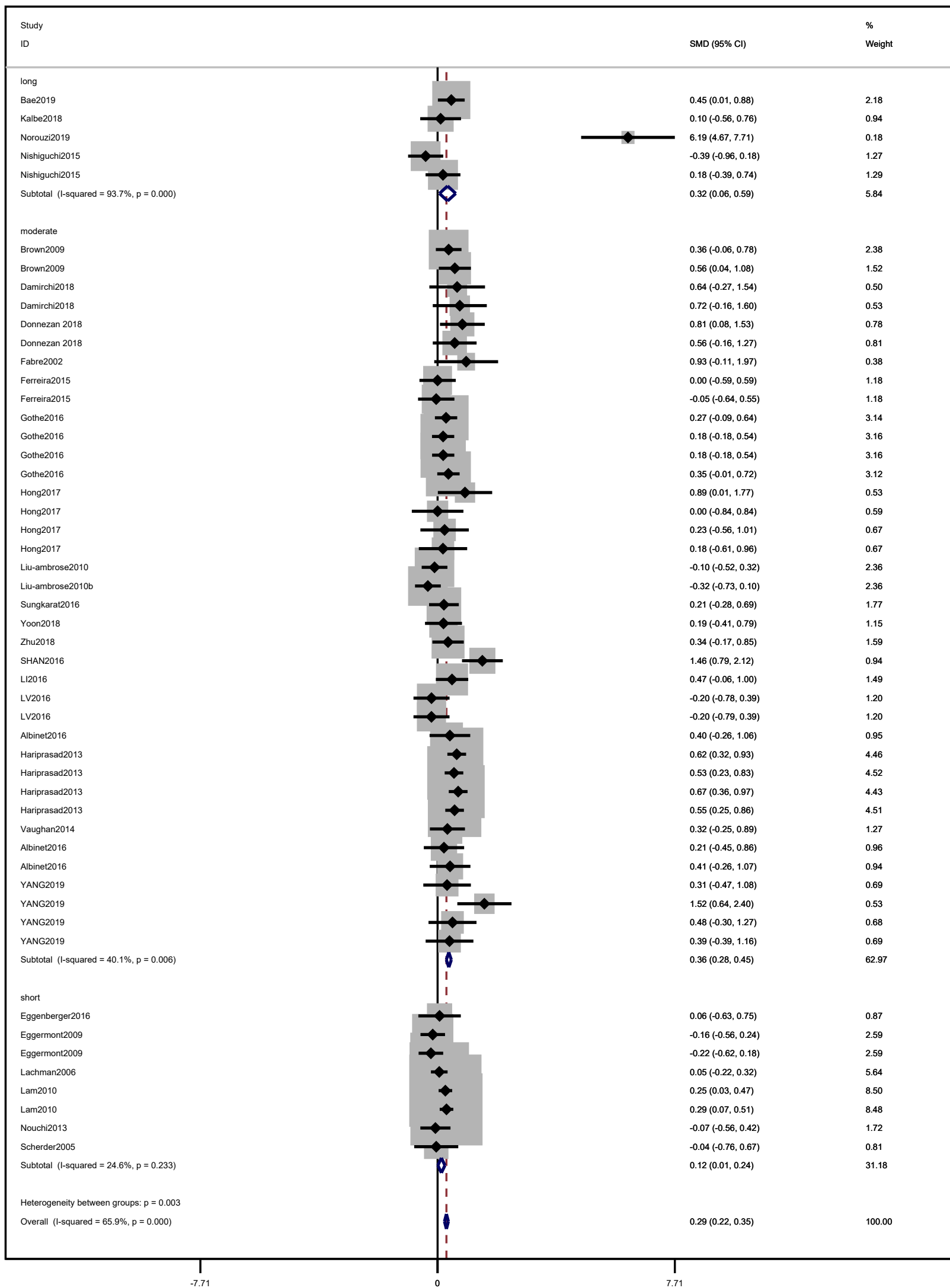

Supplement: Supplementary file 1 — Additional file 1. [file 11556_2021_272_MOESM1_ESM.zip › 11556_2021_272_MOESM1_ESM/subgroup-daration.pdf]

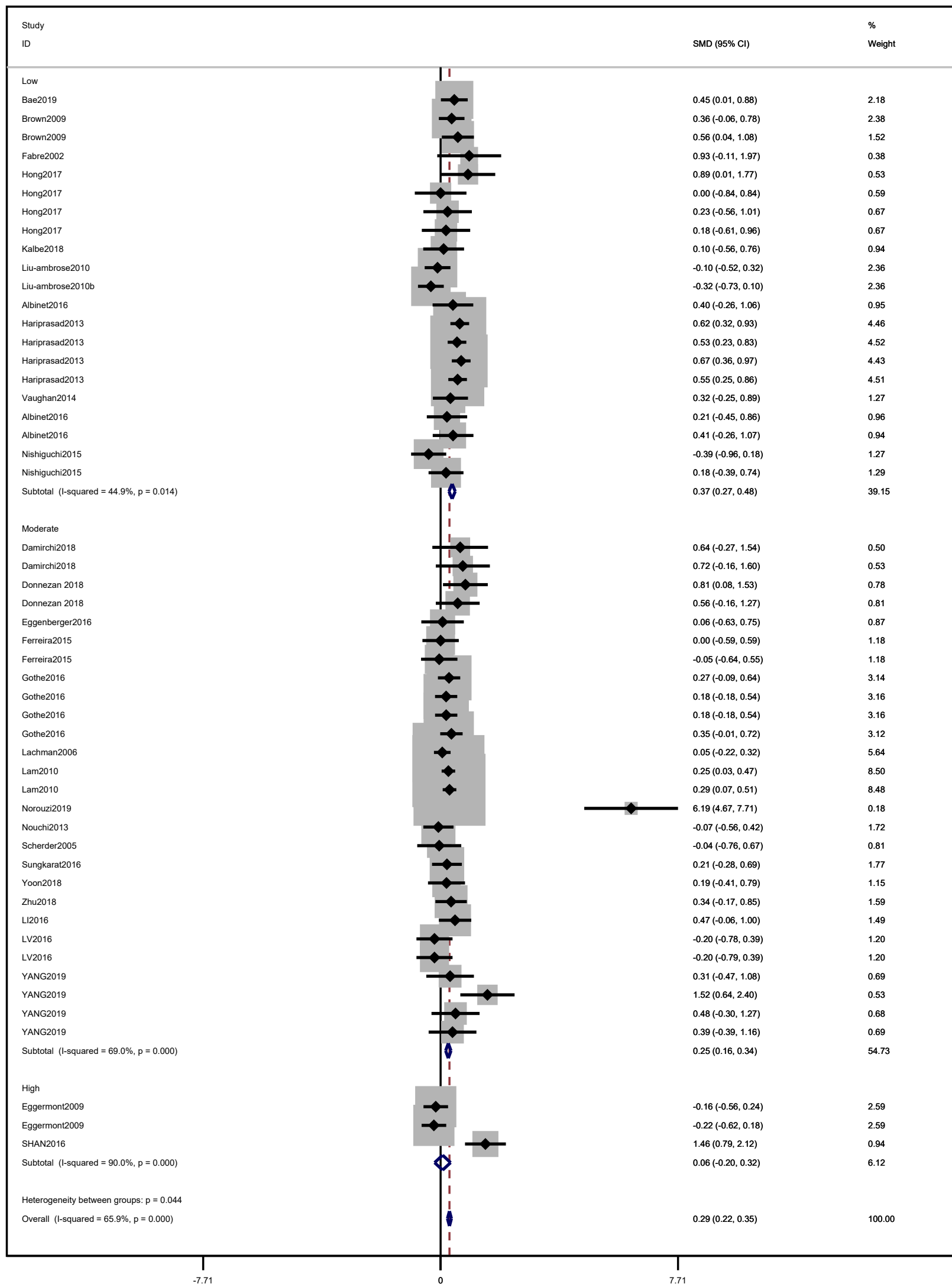

Supplement: Supplementary file 1 — Additional file 1. [file 11556_2021_272_MOESM1_ESM.zip › 11556_2021_272_MOESM1_ESM/subgroup-frequency.pdf]

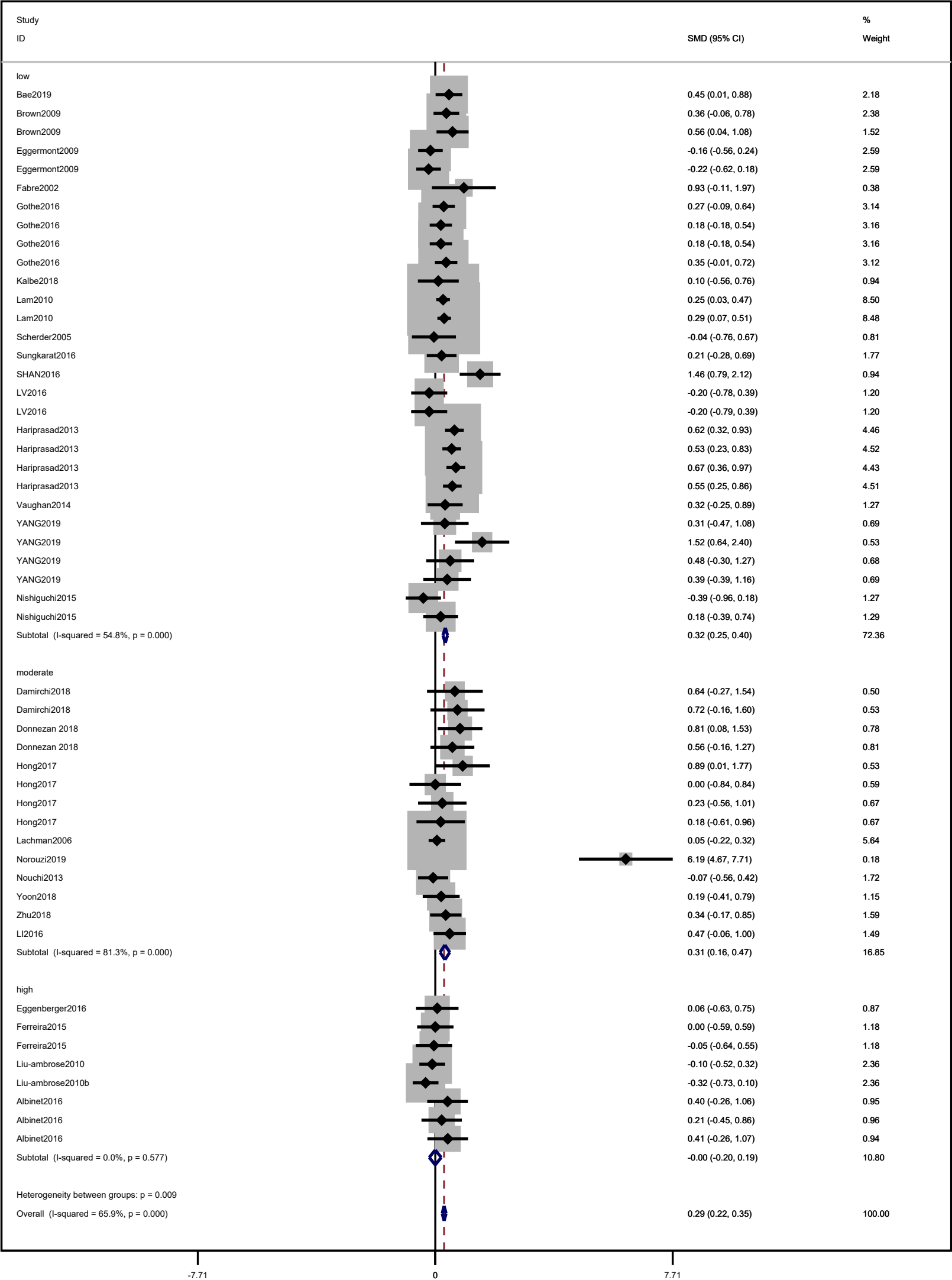

Supplement: Supplementary file 1 — Additional file 1. [file 11556_2021_272_MOESM1_ESM.zip › 11556_2021_272_MOESM1_ESM/subgroup-intensity.pdf]

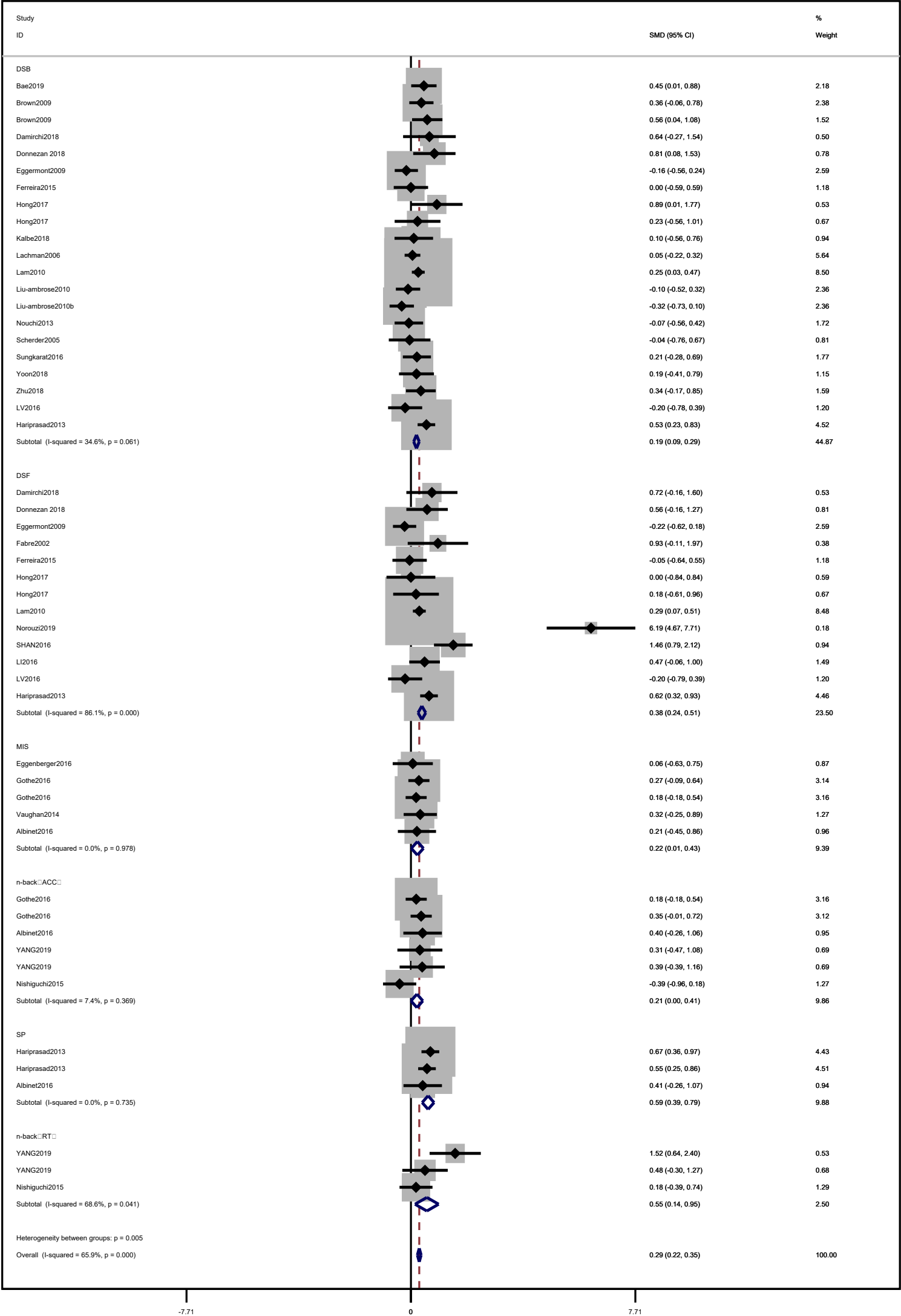

subgroup forest plot - measurement

Supplement: Supplementary file 1 — Additional file 1. [file 11556_2021_272_MOESM1_ESM.zip › 11556_2021_272_MOESM1_ESM/subgroup-measurement.pdf]

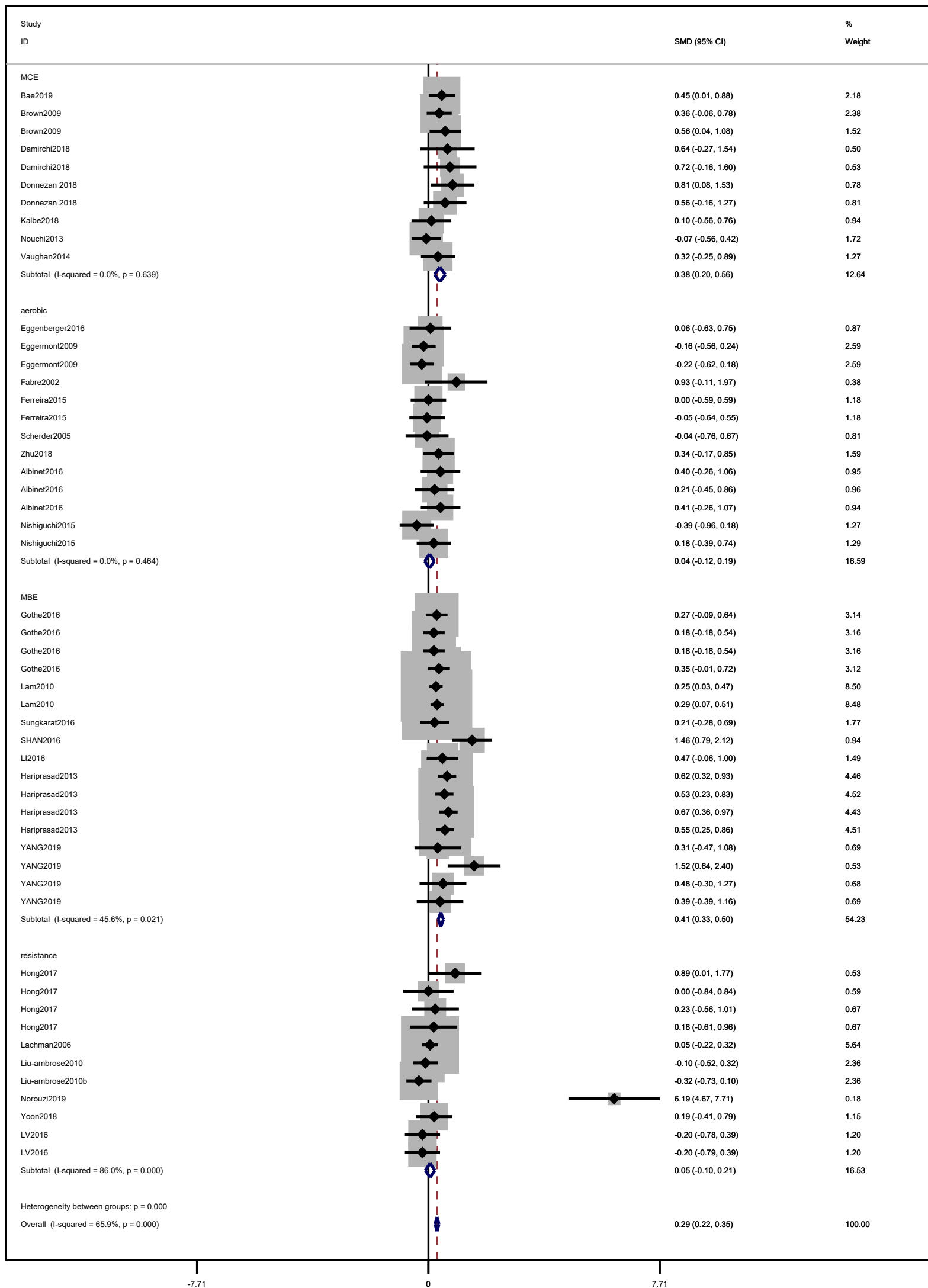

subgroup forest plot - type

Supplement: Supplementary file 1 — Additional file 1. [file 11556_2021_272_MOESM1_ESM.zip › 11556_2021_272_MOESM1_ESM/subgroup-type.pdf]
